# Ochronotic arthropathy: skeletal manifestations and orthopaedic treatment

**DOI:** 10.1530/EOR-2023-0112

**Published:** 2025-02-03

**Authors:** Khaled Hamed Salem, Alyaa Diaa Elmoghazy

**Affiliations:** ^1^ Department of Orthopaedic Surgery, Faculty of Medicine, Cairo University, Cairo, Egypt; ^2^ Department of Orthopaedic Surgery, RWTH University Aachen, Aachen, Germany; ^3^ Faculty of Medicne, Minia University, Minia, Egypt

**Keywords:** ochronosis, ochronotic arthropathy, arthroplasty, alkaptonuria, black joints

## Abstract

- Alkaptonuria is an extremely rare disorder of tyrosine metabolism caused by an autosomal recessive enzymatic deficiency of homogentisic acid (HGA) oxidase, causing its accumulation in collagenous structures, especially in hyaline cartilage.
- It is characterized by a triad of homogentisic aciduria, bluish-black discoloration of connective tissues (ochronosis) and arthropathy of the spine and large weight-bearing joints.
- Several clinical manifestations were described including coronary and valvular calcification, aortic stenosis, limited chest expansion, and renal, urethral and prostate calculi as well as ocular and cutaneous pigmentation.
- Skeletal affection usually presents as spondylotic changes of the spine. The knee is the most common peripheral joint to be involved. Enthesopathy or tendon ruptures may occur, and reduced bone density is not unusual.
- A low-protein diet and ascorbic acid may reduce HGA levels. Nitisinone can safely and effectively reduce HGA production and urinary excretion.
- In severe ochronotic arthropathy, joint arthroplasty can offer reliable pain relief and excellent functional outcomes. Cementless fixation is successful in young patients.

## Introduction

Alkaptonuria is an extremely rare autosomal recessive metabolic disease caused by deficiency of homogentisic acid (HGA) 1,2-dioxygenase, affecting one in 250,000–1,000,000 live births (1, 2, 3, 4, 5). This enzyme normally changes HGA to maleylacetoacetic acid, and its deficiency results in accumulation of HGA and its oxidized product, benzoquinone acetic acid, in collagenous structures and excessive renal excretion, causing urinary discoloration on standing or alkalinization, hence the name alkaptonuria (2, 6, 7). It is characterized by a triad of homogentisic aciduria, ochronosis and progressive degeneration of all affected structures with widespread joint arthritis (ochronotic arthropathy (OcA)) (4, 5).

Ochronosis is the deposition of bluish-black pigmented metabolic by-products that look microscopically ochre (yellow in Greek) in connective tissues, especially in hyaline cartilage (2, 8). In addition to ocular and cutaneous pigmentation, affected individuals can suffer a wide spectrum of manifestations (9). Cardiovascular involvement usually presents with coronary and valvular calcifications, sometimes with aortic stenosis (4, 10, 11). Stiffness of the costal cartilage can cause limited chest expansion with dyspnea (12). In the genitourinary tract, renal, urethral and prostate calculi are common (2, 4, 9). Systemic metabolic affection reduces bone density causing osteopenia and osteoporosis (7). Ochronotic tendinopathy usually affects large tendons (e.g. Achilles and patellar tendons), causing enthesopathy and sometimes spontaneous rupture (7).

OcA is the most common complication of alkaptonuria (4). It usually remains asymptomatic until the fourth decade of life because of reduced renal clearance of HGA with age (2, 7, 13). Thereafter, the condition progresses rapidly with joint pain, swelling, limited range of motion and stiffness causing disability and reduced quality of life in affected individuals (4, 6, 7, 13). OcA usually involves the cervical, thoracic and lumbosacral spine causing disk degeneration and spondylotic changes (7, 12). The knee is the most common peripheral joint to be affected (7, 14, 15, 16).

## Historical review

The first verified case of ochronosis was described in an Egyptian mummy (Harwa) from 1500 B.C. (17). Scribonius in 1584 reported a boy who passed urine as black as ink (18). Boedeker in 1859 first used the term “Alcapton” to describe a second urinary reducing substance in a patient with glycosuria on account of its behavior with alkali (19). The substance was identified in January 1891 as 2,5-dihydroxyphenylacetic acid or HGA by Wolkow & Baumann (20). Virchow called the condition ochronosis (yellow disease) in October 1866 because the pigment accumulated looks yellow under microscopy (21). Albrecht (22) was the first to recognize that ochronosis and alkaptonuria represent different aspects of the same disease in 1902. In the same year, Garrod in London identified the hereditary nature of the disease (23). In June 1908, he described alkaptonuria in his Croonian lectures as the first inborn error of metabolism that obeys the Mendelian principles of autosomal recessive inheritance in humans (24). Neubauer (25) mapped the complete tyrosine-degradation pathway by 1909. Several years later, the specific hepatic enzyme defect in alkaptonuria was demonstrated to be HGA-oxidase deficiency, one of six enzymes required for the catabolism of the aromatic amino acids phenylalanine and tyrosine (26).

## Etiopathology

Several mechanisms for the development of ochronotic arthritis have been described. Hamdi and coworkers proposed that benzoquinone acetic acid inhibits lysine hydroxylase enzyme and thereby reduces cross-linkage of collagen fibers (13). This enhances fiber vulnerability to stress and shearing injury causing connective tissue failure with cartilage fragmentation (13). Synovial adherence of these cartilage fragments may cause inflammation, fibrosis, loose body formation or chondromatosis (27). Taylor and coworkers attributed ochronotic arthritis to altered mechanical properties of the pigmented hyaline cartilage that becomes weak and brittle, loses elasticity and develops poor resistance to mechanical strain (8). Furthermore, it has been shown that HGA oxidation produces free oxygen radicals, which may induce inflammatory, degenerative and amyloidogenic changes (7, 28). On the cellular level, this oxidative stress was recently proved to affect human osteoblastic functionality and induce autophagy alterations leading to chondroptosis in human chondrocytes (29, 30). In addition, pigment deposits may initiate aggressive osteoclastic resorption underneath hyaline cartilage, causing complete loss of the subchondral plate (8). Ochronotic pigments deposited in bone impair mineralization of the newly formed osteoid matrix with decreased bone mineral density and an increased risk of fragility fractures (7).

## Clinical presentation

Unlike rheumatoid arthritis, which affects the small joints of the hands and feet, OcA predominantly involves the spine and large weight-bearing joints and in contrast to ankylosing spondylitis, the sacroiliac joints are relatively spared and bamboo spine, annular ossification and syndesmophytes do not occur (7, 31). Patients with ochronotic spine disease typically present with back pain and stiffness, eventual loss of lordosis and exaggeration of thoracic kyphosis (7). Ochronotic spondylarthropathy is claimed to be more severe in HLA-B27-positive individuals (32). Radiologically, spine affection is associated with disk herniation, extensive multilevel calcification of ochronotic intervertebral disks and vacuum phenomena (7). Further degenerative changes may cause spinal stenosis with myelopathy. Affection of the large weight-bearing joints occurs several years after spinal involvement. The knee is the most commonly affected peripheral joint in up to 64% of cases (7). Upper limb involvement is extremely rare. The radiological features of peripheral joint involvement include severe degenerative changes with loss of joint space and subchondral bone sclerosis, with minimal or no osteophytic changes (in contrast to osteoarthritis). Aspiration of the synovial fluid often reveals floating black particles (ground pepper sign) (33). Coexistence of ochronosis and rheumatoid arthritis, ankylosing spondylitis or chondrocalcinosis has also been reported (4).

## Treatment

Treatment options are limited in alkaptonuria (2). Conservative treatment aims at controlling and ameliorating symptoms (31). Protein restriction and ascorbic acid have been shown to reduce urinary HGA excretion with reversal of bone abnormalities (34). However, a low-protein diet is difficult to maintain in such a lifelong disease. Nitisinone (Orfadin^®^), a synthetic reversible inhibitor of 4-hydroxyphenylpyruvate dioxygenase, is now the first disease-modifying drug approved by the European Medicine Agency (EMA) in 2020 for adults with alkaptonuria. It was originally approved by the Food and Drug Administration (FDA) in 2002 for the treatment of patients with hereditary tyrosinemia type I (HT-1). Two multicenter international studies (SONIA 1 with 40 and SONIA 2 with 138 patients) proved that nitisinone causes a safe and effective reduction of urinary HGA excretion in patients with alkaptonuria up to 99.7% with significant clinical improvement (35, 36). Genovese *et al.* demonstrated that nitisinone induced changes in serological and urinary biomarkers of bone and cartilage remodeling (37). However, it has little effect on the increased serum amyloid A observed in alkaptonuria, which may cause secondary amyloidosis (38). In ochronotic arthritis, physiotherapy, pain control and nonsteroidal anti-inflammatory drugs can limit symptoms with no effect on disease progression (39). In end-stage cases of OcA, surgical intervention through total joint replacement represents the best treatment available (6, 31).

## Authors’ own experience

**Figure 1:**
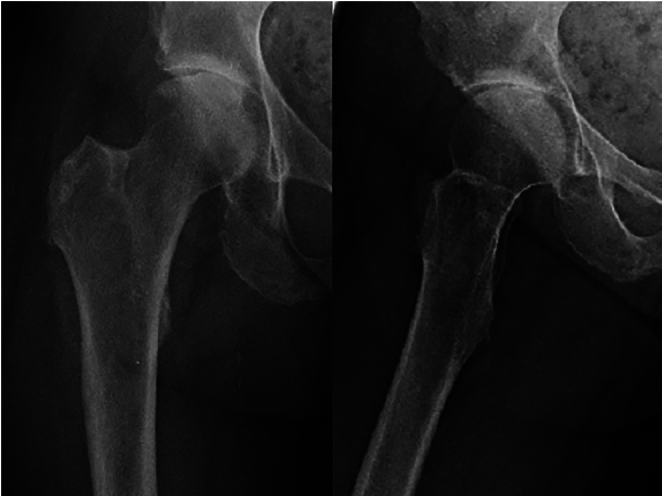
Preoperative X-rays showing arthritis of the right hip.

**Figure 2:**
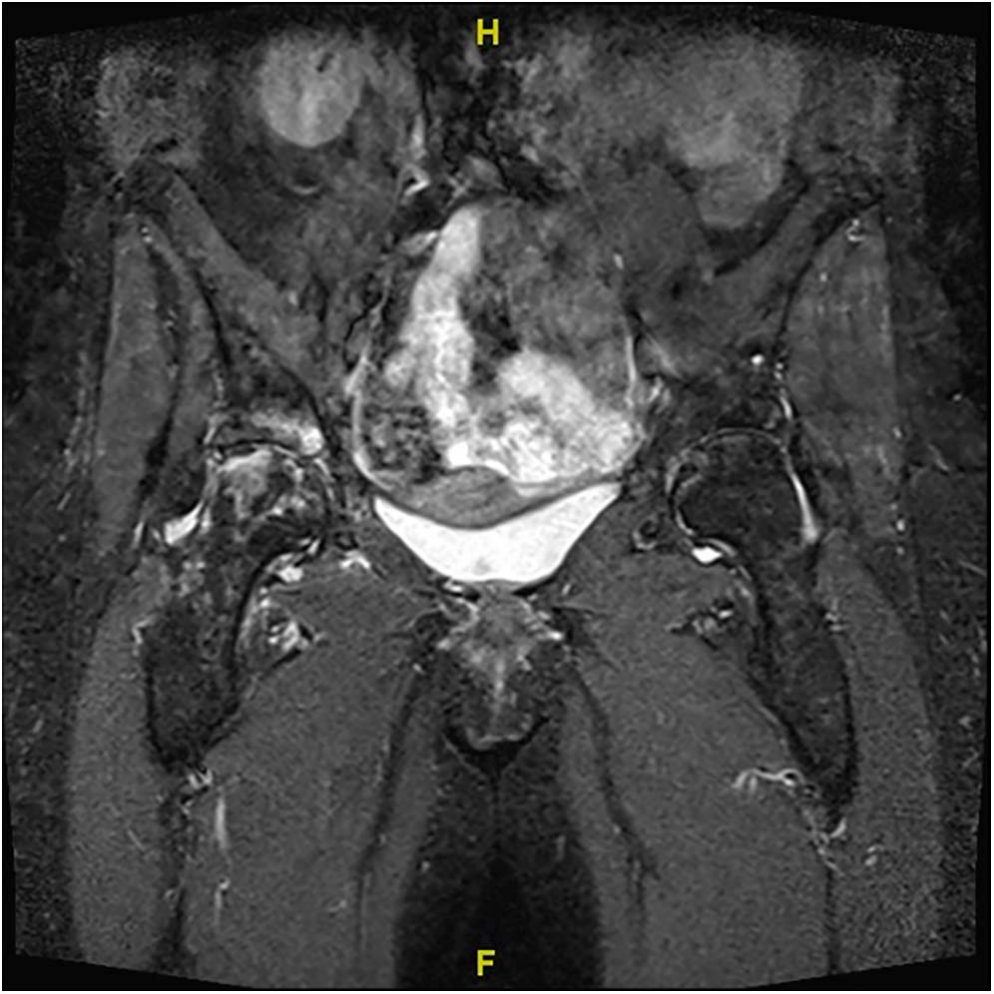
MRI image of bone and cartilage destruction of the right hip.

**Figure 3:**
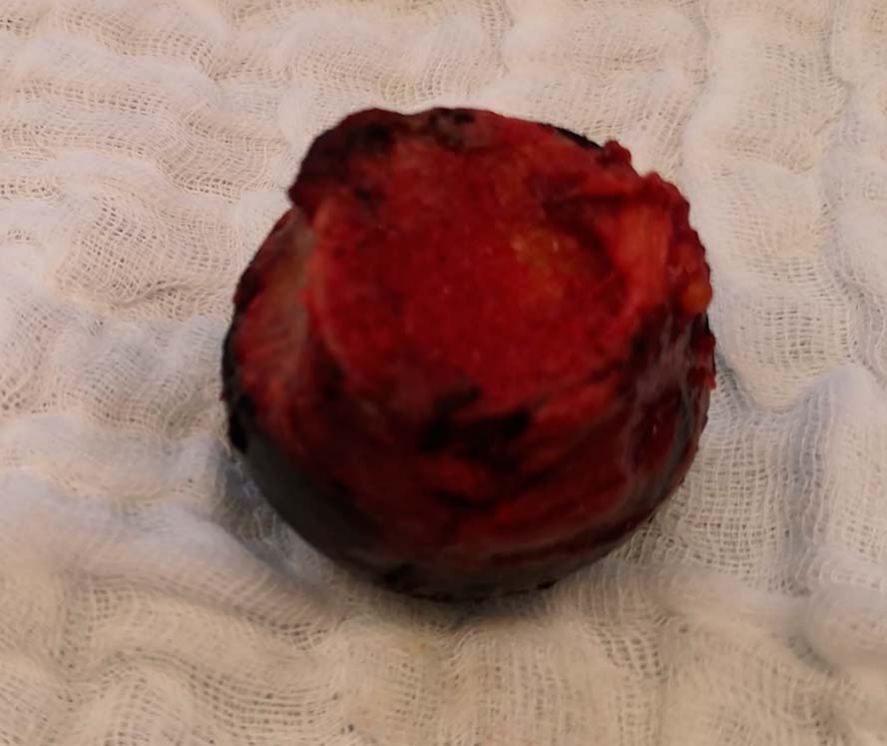
The resected femoral head showing deep black discoloration.

**Figure 4:**
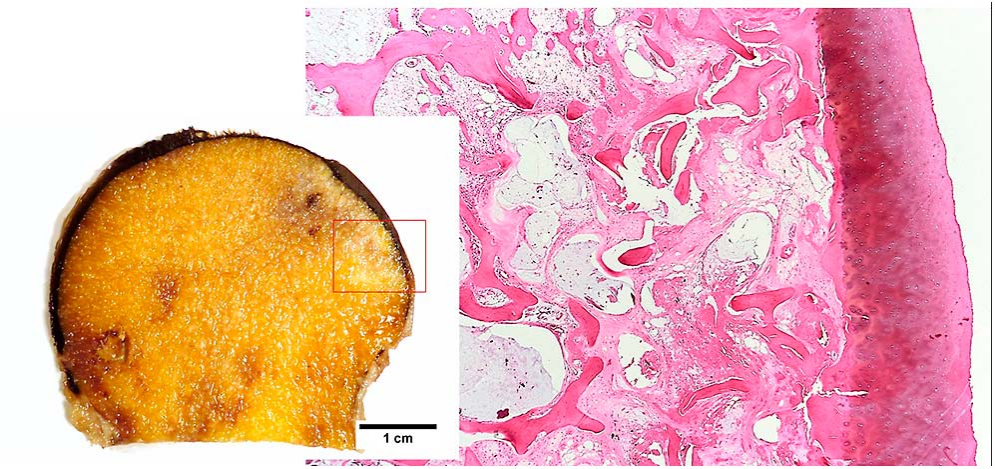
Gross and microscopic image of ochronotic arthropathy.

**Figure 5:**
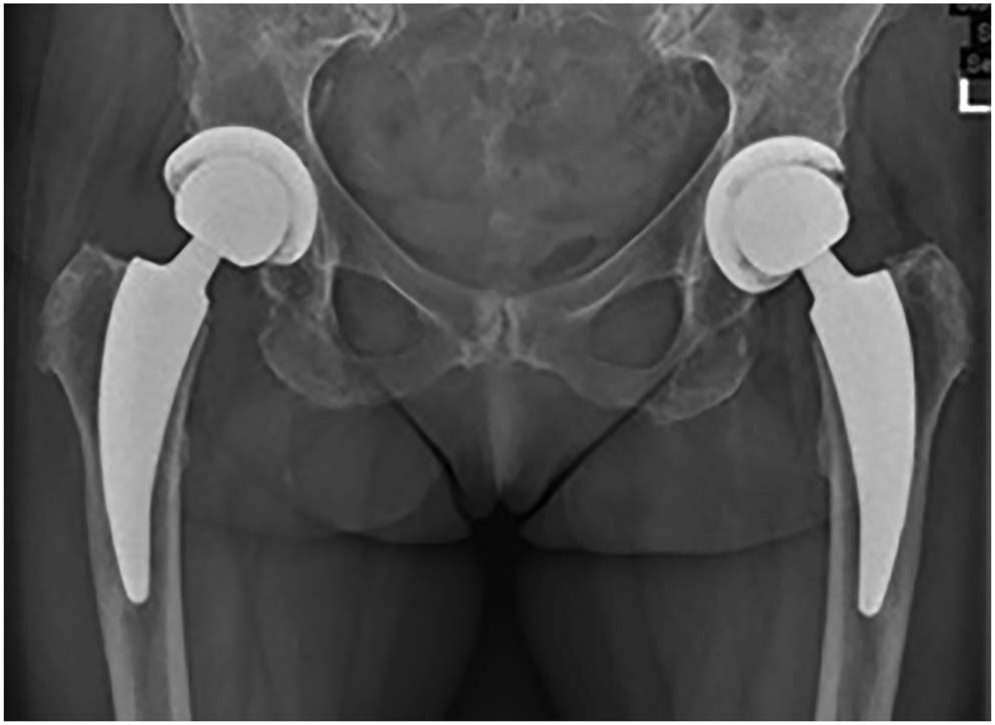
Pelvic view showing bilateral cementless total hip replacement with short stems.

**Figure 6:**
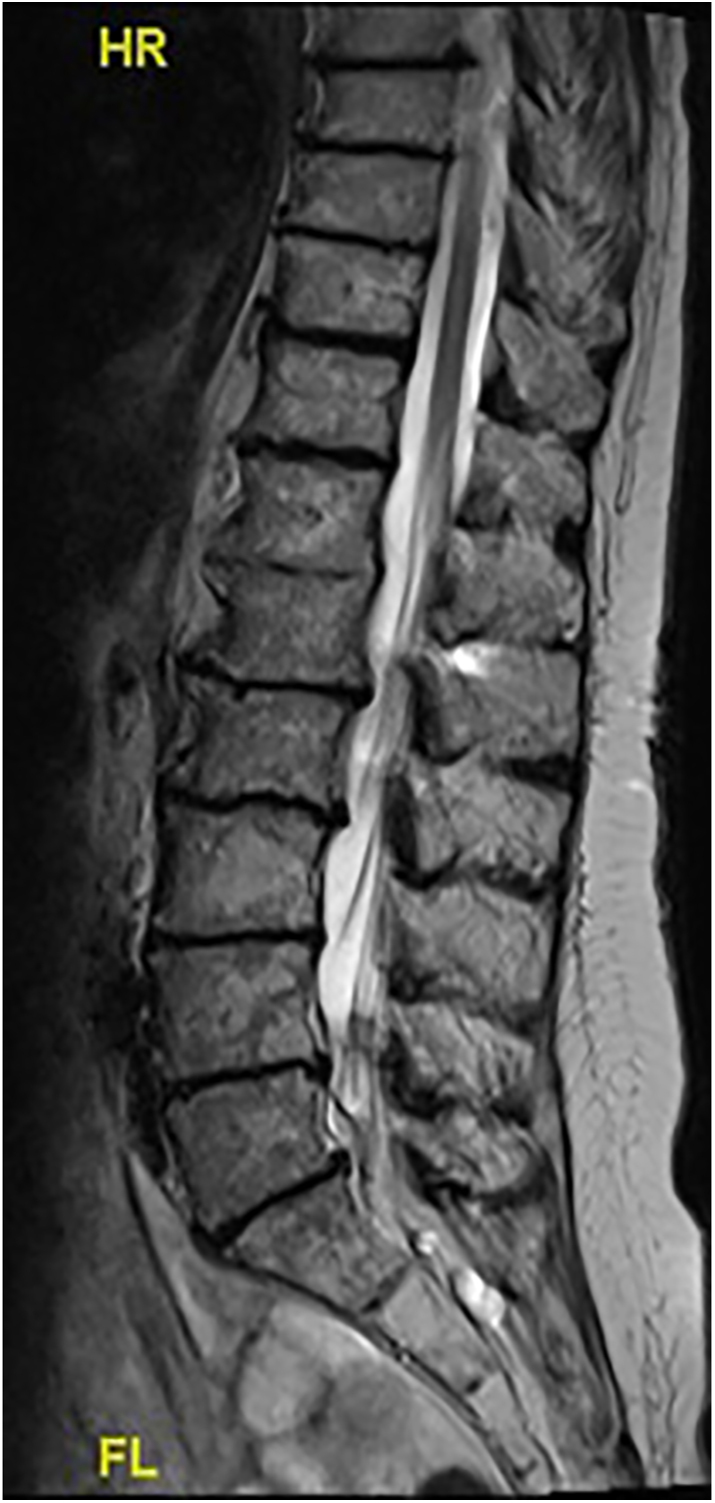
Advanced multisegmental spondylotic changes of the spine.

A middle-aged female patient suffering from polyarticular ochronotic involvement was treated in 2016 at the age of 53 years with bilateral anatomic shaftless total shoulder arthroplasty (Eclipse^TM^, Arthrex, USA). Four years later, she presented with severe right-sided hip pain with limited weight-bearing. Radiologic examination revealed advanced narrowing of the joint space, and a pelvic MRI showed osteonecrosis of the right femoral head with advanced hip arthritis (Figs 1 and 2). She was admitted to the hospital due to intractable pain not responding to conservative treatment and was treated in 2020 using cementless total hip replacement with a short-stem (ArtiQo GmbH, Germany) through a minimally invasive posterior approach. Intraoperatively, the deep black discoloration of the joint, femoral head and synovium with complete separation of the articular cartilage was obvious (Fig. 3). OcA was then confirmed by histopathologic examination (Fig. 4). After finishing her rehabilitation, the patient presented six weeks postoperatively with severe groin pain and an inability to walk on the left side with radiologically evident chondrolysis of the left hip. She was again treated using minimally invasive left-sided cementless short-stem total hip replacement (Fig. 5). The intraoperative picture was similar to that of the former operation. The postoperative phases of all surgeries were uneventful. No complications were encountered during any surgery. The patient was completely pain-free and able to walk on crutches on the first postoperative day. She received daily physical therapy, gait training and range of motion exercises for the hip joint. Hip adduction, high flexion and excessive rotation were avoided to prevent prosthetic dislocation. She continued ambulatory rehabilitation after discharge, which she tolerated quite well. Walking aids were used for just 3 weeks. At the latest follow-up visit two years after surgery, the patient had an excellent functional outcome with no pain, free range of motion, total independence in daily activities and no prosthetic loosening or subsidence. Back pain with advanced multisegmental spondylotic changes was, however evident (Fig. 6).

## Discussion

To date, only few reports have described joint arthroplasty in OcA (1, 2, 3, 4, 6, 15, 16, 31, 40, 41, 42, 43, 44, 45, 46, 47, 48, 49, 50). In the largest series of 10 patients (12 hips), Pachore *et al*. (6) achieved satisfactory functional results with total hip replacement in alkaptonuric hip arthritis after up to 24 years of follow-up. Ilyas *et al.* (2) reported a case of a 45-year-old man with bilateral total hip and knee replacement because of advanced ochronotic arthritis, with no complications after 12 years. Di Marco *et al*. (40) and Araki *et al.* (41) reported similar good results after 6–7 years following bilateral total hip and knee arthroplasty for ochronotic arthritis. Demir (42) also reported on a case of alkaptonuric ochronosis with multiple joint replacements. Ye and colleagues (31) reported on bilateral total hip and right total knee arthroplasties in a patient with alkaptonuric arthritis. Fischer & Davis (4) reported on a patient who needed total joint replacement of her both knees and the right hip joint for OcA. In addition, da Silva Martins Ferreira *et al.* (43) reported on a male patient suffering from alkaptonuria since the age of 40, undergoing total replacement of his left hip and both knees done after the age of 60 years. Fernando *et al.* (1) presented a case of bilateral OcA of the hip that was successfully managed by staged bilateral total hip replacement. Saini *et al.* (15) presented a female patient with bilateral total knee arthroplasty due to OcA. Lee *et al.* (16) also described a case of bilateral knee arthroplasty in a male patient with ochronotic arthritis. Aydoğdu *et al.*  (44) reported a case of OcA treated using cementless knee arthroplasty with a 4-year follow-up. Ozmanevra *et al.* (45) also described satisfactory results two years after simultaneous bilateral cemented knee arthroplasty in a 69-year-old male patient with ochronosis. Sahoo *et al.* (46) reported on a 51-year-old male patient with bilateral knee replacement because of severe tricompartmental osteoarthritis with varus deformities and limited range of motion. Drakoulakis *et al.* (47) reported significant improvement of shoulder function after bilateral total shoulder arthroplasty in a 53-year-old man with OcA. Acar *et al.* (3) also treated a female patient with hip and knee arthroplasty due to OcA. Mazoochy and Razi (48) reported on knee and hip joint replacement in a patient with OcA. Harun *et al.*  (49) also reported on knee and hip replacement in a 60-year-old female ochronotic patient with black joints. Cebesoy *et al.* (50) treated a 46-year-old male patient with ochronosis using cementless total hip arthroplasty of the right hip due to osteoarthritis (Table 1).

**Table 1:** Case reports on total joint arthroplasty in ochronotic arthropathy.

Case reports	Patients, *n*	Age, years	Sex	Joints	FU, years
Authors’ case 2024	1	53	Female	Both hips and shoulders	2
Pachore *et al.* (6)	10	53–80 (63)	6 males; 4 females	12 hips	3–24 (16.7)
Ilyas *et al.* (2)	1	45	Male	Both hips and knees	12
Di Marco *et al.* (40)	1	50	Female	Both hips and knees	7
Araki *et al.* (41)	1	56	Male	Both hips and knees	6
Demir (42)	1	70	Male	Both hips and knees	14
Ye *et al.* (31)	1	64	Female	Both hips and right knee	3
Fischer & Davis (4)	1	69	Female	Both knees and right hip	5–7
da Silva *et al.* (43)	1	67	Male	Left hip and both knees	0.5–5
Fernando *et al.* (1)	1	69	Female	Both hips	1.5
Saini *et al.* (15)	1	52	Female	Both knees	2
Lee *et al.* (16)	1	54	Male	Both knees	2
Aydoğdu *et al.* (44)	1	48	Male	Both knees	4
Ozmanevra *et al.* (45)	1	69	Male	Both knees	2
Sahoo *et al.* (46)	1	51	Male	Both knees	2.3
Drakoulakis *et al.* (47)	1	53	Male	Both shoulders	3
Acar *et al.* (3)	1	62	Female	Left hip and right knee	1.5
Mazoochy & Razi (48)	1	57	Female	Right hip and right knee	2
Harun *et al.* (49)	1	60	Female	Right hip and left knee	Unknown
Cebesoy *et al.* (50)	1	46	Male	Right hip	0.5

FU, follow-up.

The above-mentioned own case confirms the excellent outcomes of cementless arthroplasty in OcA. The presented patient underwent bilateral total shoulder and hip replacements with an excellent functional outcome with full mobility and no pain or complications after two years of the last surgery. This compares favorably to former case reports that showed excellent short-term results in patients with OcA treated with joint arthroplasty. Pachore *et al.* suggested a total synovectomy, which we also did in our procedures (6). Total resection of the joint capsule, as recommended by Cebesoy *et al.*, was, however, avoided due to stability issues with the posterior hip approach we used (50). Difficulties in achieving primary stability of the cementless implants were not encountered despite reduced quality of the affected bone, and no subsidence of the short stems was observed. This compares favorably to the long-term results reported by Ilyas *et al.* and Pachore *et al*. with cementless total hip replacement (2, 6).

## Conclusion

In conclusion, alkaptonuric ochronosis is an ultra-rare, disabling, autosomal recessive metabolic disease causing severe polyarticular arthropathy with cartilage and bone destruction in relatively young patients. Total joint replacement with bone-sparing cementless implants can yield consistent pain relief and satisfactory functional recovery in patients with end-stage OcA.

## ICMJE Statement of Interest

The authors declare that there is no conflict of interest that could be perceived as prejudicing the impartiality of the work reported.

## Funding Statement

This work did not receive any specific grant from any funding agency in the public, commercial or not-for-profit sector.

## References

[1] Fernando OSF, Supreeth N, David GD, et al. An unusual case of bilateral ochronotic arthropathy of the hip successfully managed by a staged bilateral total hip replacement – an insight with a surgical note. J Orthop Case Rep 2018 8 11. (10.13107/jocr.2250-0685.1136) PMC6343567 30687653

[2] Ilyas I, Kashif S, Algashiri MF, et al. Long-term follow-up of bilateral hip and knee arthroplasty secondary to ochronotic arthropathy. Arthroplast Today 2020 6 214–219. (10.1016/j.artd.2020.01.012) 32577465 PMC7303484

[3] Acar MA, Erkocak OF, Aydin BK, et al. Patients with black hip and black knee due to ochronotic arthropathy: case report and review of literature. Oman Med J 2013 28 448–449. (10.5001/omj.2013.124) 24223251 PMC3815858

[4] Fisher AA & Davis MW Alkaptonuric ochronosis with aortic valve and joint replacements and femoral fracture: a case report and literature review. Clin Med Res 2004 2 209–215. (10.3121/cmr.2.4.209) 15931360 PMC1069096

[5] Janocha S, Wolz W, Srsen S, et al. The human gene for alkaptonuria (AKU) maps to chromosome 3q. Genomics 1994 19 5–8. (10.1006/geno.1994.1003) 8188241

[6] Pachore JA, Shah VI, Upadhyay S, et al. Primary hip arthroplasty for the treatment of alkaptonuric hip arthritis: 3- to 24-year follow-ups. Arthroplasty 2019 1 8. (10.1186/s42836-019-0010-8) 35240771 PMC8796533

[7] Wu K, Bauer E, Myung G, et al. Musculoskeletal manifestations of alkaptonuria: a case report and literature review. Eur J Rheumatol 2019 6 96–99. (10.5152/eurjrheum.2018.18116) PMC6467321 30451653

[8] Taylor AM, Boyde A, Wilson PJ, et al. The role of calcified cartilage and subchondral bone in the initiation and progression of ochronotic arthropathy in alkaptonuria. Arthritis Rheum 2011 63 3887–3896. (10.1002/art.30606) 22127706

[9] Van Offel JF, De Clerck LS, Francx LM, et al. The clinical manifestations of ochronosis: a review. Acta Clin Belg 1995 50 358–362. (10.1080/17843286.1995.11718475) 8571731

[10] El-Sayed Ahmed MM, Hussain O, Ott DA, et al. Severe aortic valve stenosis due to alkaptonuric ochronosis. Semin Cardiothorac Vasc Anesth 2017 21 364–366. (10.1177/1089253217720284) 28709382

[11] Steger CM Aortic valve ochronosis: a rare manifestation of alkaptonuria. BMJ Case Rep 2011 2011 bcr0420114119. (10.1136/bcr.04.2011.4119) PMC3149459 22689837

[12] Collins E & Hand R Alkaptonuric ochronosis: a case report. AANA J 2005 73 41–46. 15727283

[13] Hamdi N, Cooke TD & Hassan B Ochronotic arthropathy: case report and review of the literature. Int Orthop 1999 23 122–125. (10.1007/s002640050325) 10422033 PMC3619804

[14] G S, John JT, Nair DS, et al. Ochronotic surprise during total knee replacement! A case report. J Orthop Case Rep 2021 11 49–52. (10.13107/jocr.2021.v11.i10.2464) PMC8930298 35415103

[15] Saini MK, Reddy NR, Reddy PJ, et al. Clinical and surgical insights on bilateral total knee arthroplasty in ochronotic arthropathy: a case-based review. J Orthop Case Rep 2021 11 30–34. (10.13107/jocr.2021.v11.i12.2554) PMC8930374 35415137

[16] Lee WC, Tan TL & Chan YH Total knee arthroplasty in ochronosis arthropathy: a case report and systematic review. Case Rep Orthop 2019 2019 1–8. (10.1155/2019/1871856) PMC6803722 31687244

[17] Stenn FF, Milgram JW, Lee SL, et al. Biochemical identification of homogentisic acid pigment in an ochronotic egyptian mummy. Science 1977 197 566–568. (10.1126/science.327549) 327549

[18] Scribonius GA. De inspectione urinarum. Germany: Lemgo 1584 **50**.

[19] Boedeker CW Ueber das Alcapton; ein neuer Beitrag zur Frage: welche Stoffe des Harns können Kupferreduction bewirken? Z Rat Med 1859 7 130–145.

[20] Wolkow M & Baumann E Ueber das Wesen der Alkaptonurie. Zeitschr Physiol Chem 1891 15 228–285.

[21] Virchow R Ein Fall von allgemeiner Ochronose der Knorpel und knorpelähnlichen Theile. Virchows Arch Pathol Anat Physiol 1866 37 212–219. (10.1007/bf01935634)

[22] Albrecht H. Ueber Ochronose. Z Heilk 1902 23 366–378.

[23] Garrod AE The incidence of alkaptonuria: a study in chemical individuality. Lancet 1902 160 1616–1620. (10.1016/s0140-6736(01)41972-6)

[24] Garrod AE The Croonian lectures on inborn errors of metabolism. Lancet 1908 172 1–7. (10.1016/s0140-6736(01)78482-6)

[25] Neubauer O Über den Abbau der Aminosäuren im gesunden und kranken Organismus. Dtsch Arch Klin Med 1909 95 211–256.

[26] La Du BN, Seegmiller JE, Laster L, et al. Alcaptonuria and ochronotic arthritis. Bull Rheum Dis 1958 8 163–164. 13523305

[27] Ventura-Ríos L, Hernández-Díaz C, Gutiérrez-Pérez L, et al. Ochronotic arthropathy as a paradigm of metabolically induced degenerative joint disease. A case-based review. Clin Rheumatol 2016 35 1389–1395. (10.1007/s10067-014-2557-7) 24647979

[28] Milella MS, Geminiani M, Trezza A, et al. Alkaptonuria: from molecular insights to a dedicated digital platform. Cells 2024 13 1072. (10.3390/cells13121072) 38920699 PMC11201470

[29] Schiavone ML, Millucci L, Bernardini G, et al. Homogentisic acid affects human osteoblastic functionality by oxidative stress and alteration of the Wnt/β-catenin signaling pathway. J Cell Physiol 2020 235 6808–6816. (10.1002/jcp.29575) 31989660

[30] Galderisi S, Milella MS, Rossi M, et al. Homogentisic acid induces autophagy alterations leading to chondroptosis in human chondrocytes: implications in alkaptonuria. Arch Biochem Biophys 2022 717 109137. (10.1016/j.abb.2022.109137) 35090868

[31] Ye C-Y, Xue D-T, Chen X, et al. Multiple arthroplasty in a patient with alkaptonuric arthritis. Chin Med J 2015 128 2404–2405. (10.4103/0366-6999.163395) 26315091 PMC4733810

[32] Borman P, Bodur H & Ciliz D Ochronotic arthropathy. Rheumatol Int 2002 21 205–209. (10.1007/s00296-002-0175-1) 11958438

[33] Hunter T, Gordon DA & Ogryzlo MA The ground pepper sign of synovial fluid: a new diagnostic feature of ochronosis. J Rheumatol 1974 1 45–53. 4459470

[34] Morava E, Kosztolanyi G, Engelke UF, et al. Reversal of clinical symptoms and radiographic abnormalities with protein restriction and ascorbic acid in alkaptonuria. Ann Clin Biochem 2003 40 108–111. (10.1258/000456303321016268) 12542920

[35] Ranganath LR, Milan AM, Hughes AT, et al. Suitability of nitisinone in alkaptonuria 1 (SONIA 1): an international, multicentre, randomised, open-label, no-treatment controlled, parallel-group, dose-response study to investigate the effect of once daily nitisinone on 24-h urinary homogentisic acid excretion in patients with alkaptonuria after 4 weeks of treatment. Ann Rheum Dis 2016 75 362–367. (10.1136/annrheumdis-2014-206033) 25475116

[36] Ranganath LR, Psarelli EE, Arnoux JB, et al. Efficacy and safety of once-daily nitisinone for patients with alkaptonuria (SONIA 2): an international, multicentre, open-label, randomised controlled trial. Lancet Diabetes Endocrinol 2020 8 762–772. (10.1016/S2213-8587(20)30228-X) 32822600

[37] Genovese F, Frederiksen P, Bay-Jensen AC, et al. Nitisinone treatment affects biomarkers of bone and cartilage remodelling in alkaptonuria patients. Int J Mol Sci 2023 24 10996. (10.3390/ijms241310996) 37446173 PMC10341572

[38] Braconi D, Geminiani M, Psarelli EE, et al. Effects of nitisinone on oxidative and inflammatory markers in alkaptonuria: results from SONIA1 and SONIA2 studies. Cells 2022 11 3668. (10.3390/cells11223668) 36429096 PMC9688277

[39] Gil JA, Wawrzynski J & Wayasz GR Orthopedic manifestations of ochronosis: pathophysiology, presentation, diagnosis, and management. Am J Med 2016 129 536e1–6. (10.1016/j.amjmed.2016.01.010) 26844634

[40] Di Marco M, De Martinis SE, Truzzi M, et al. Polyarticular ochronotic arthritis: a case report. Case Rep Orthop Res 2020 2 1–13. (10.1159/000500236)

[41] Araki K, Sudo A, Hasegawa M, et al. Devastating ochronotic arthropathy with successful bilateral hip and knee arthroplasties. J Clin Rheumatol 2009 15 138–140. (10.1097/rhu.0b013e31819e6b41) 19300282

[42] Demir S Alkaptonuric ochronosis: a case with multiple joint replacement arthroplasties. Clin Rheumatol 2003 22 437–439. (10.1007/s10067-003-0760-z) 14677022

[43] da Silva Martins Ferreira AM, Lima Santos F, Castro Costa AM, et al. Knee osteoarthrosis secondary to ochronosis – clinical case. Rev Bras Ortop 2014 49 675–680. (10.1016/j.rboe.2013.11.001) 26229881 PMC4487493

[44] Aydoğdu S, Cullu E, Ozsoy MH, et al. Cementless total knee arthroplasty in ochronotic arthropathy: a case report with a 4-year follow-up. J Arthroplasty 2000 15 539–543. (10.1054/arth.2000.4228) 10884219

[45] Ozmanevra R, Güran O, Karatosun V, et al. Total knee arthroplasty in ochronosis: a case report and critical review of the literature. Eklem Hastalik Cerrahisi 2013 24 169–172. (10.5606/ehc.2013.36) 24191883

[46] Sahoo MM, Mahapatra SK, Sethi GC, et al. Patellar ligament rupture during total knee arthroplasty in an ochronotic patient. Acta Orthop Traumatol Turc 2014 48 367–370. (10.3944/aott.2014.3245) 24901931

[47] Drakoulakis E, Varvitsiotis D, Psarea G, et al. Ochronotic arthropathy: diagnosis and management: a critical review. Am J Orthop (Belle Mead NJ) 2012 41 80–83. 22482092

[48] Mazoochy H & Razi M. Knee and hip joint replacement surgery in a patient with ochronotic arthropathy: surgical tips. Arch Bone Jt Surg 2018 6 577–581. 30637315 PMC6310191

[49] Harun M, Hayrettin Y, Serhat M, et al. A rare cause of arthropathy: an ochronotic patient with black joints. Int J Surg Case Rep 2014 5 554–557. (10.1016/j.ijscr.2014.06.015) 25034257 PMC4147659

[50] Kaya O, Isik M, Subasi M, et al. Total hip replacement for an ochronotic patient: a technical trick. Am J Case Rep 2014 15 27–30. (10.12659/ajcr.890008) 24459540 PMC3899173

